# Supplementary figures and images for: CsrA selectively modulates sRNA-mRNA regulator outcomes
Source: Front Mol Biosci. 2023 Nov 21;10:1249528. doi: 10.3389/fmolb.2023.1249528 (PMC10729762; doi:10.3389/fmolb.2023.1249528)

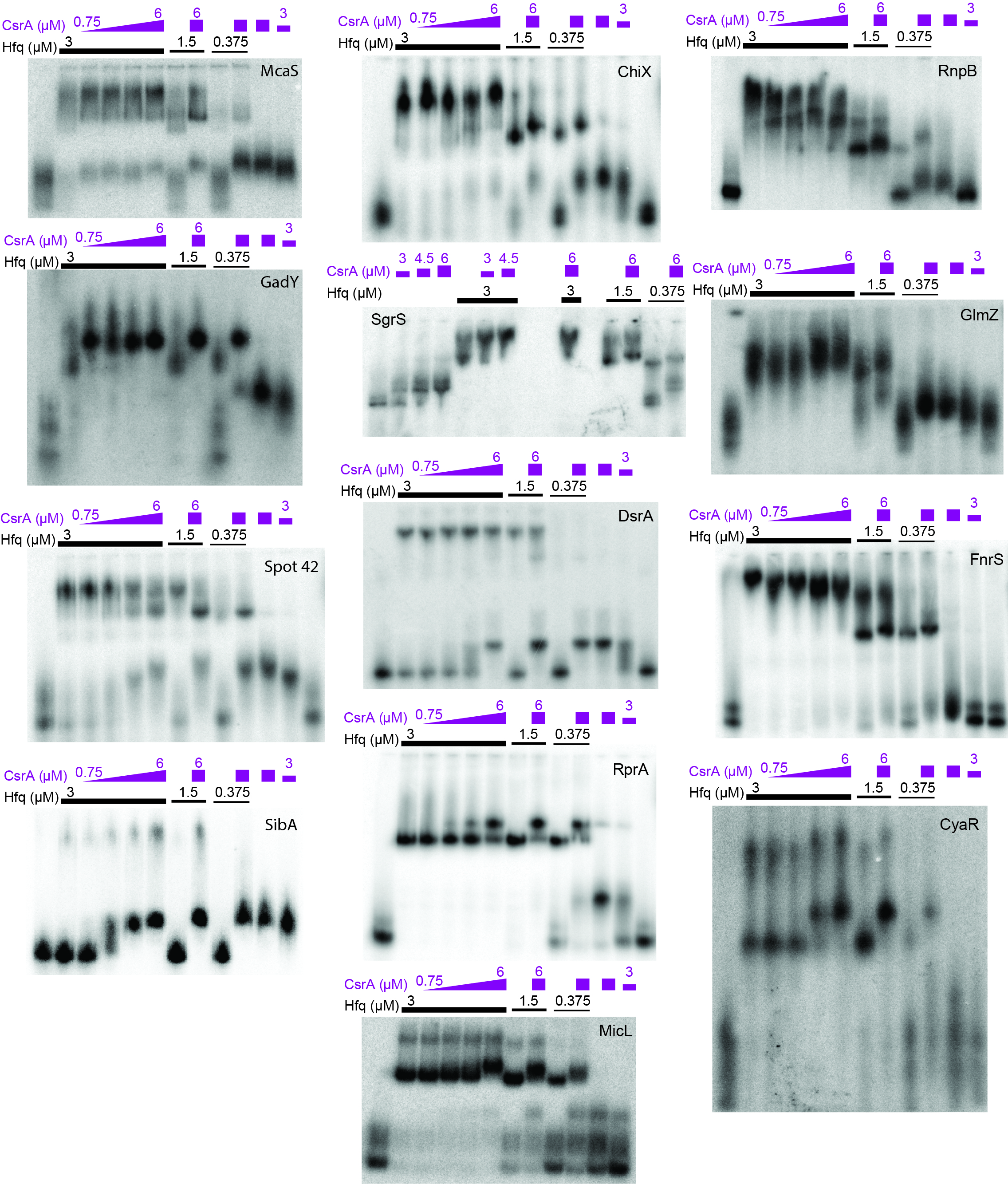

Supplement: Supplementary file 1 [file Image3.JPEG]

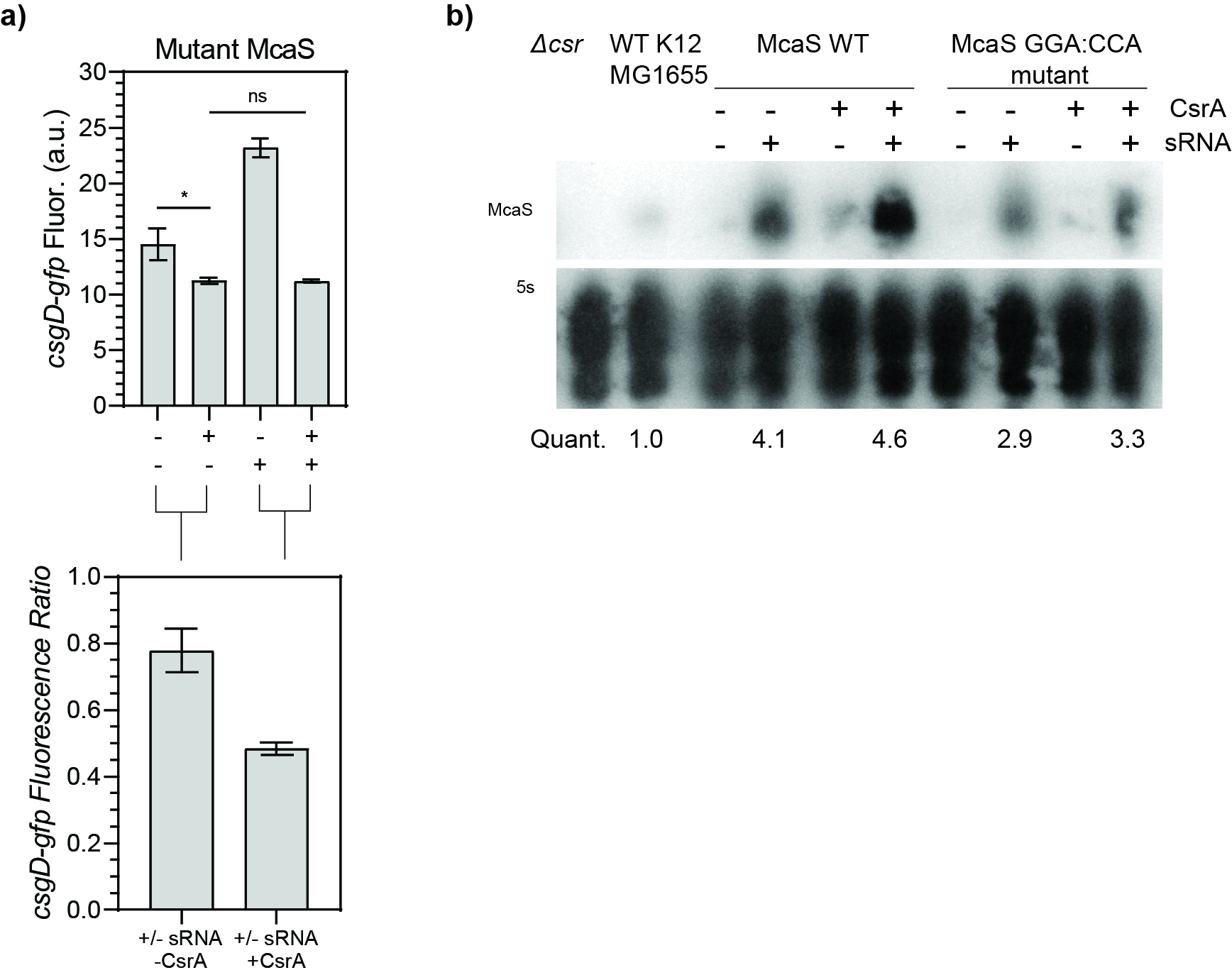

Supplement: Supplementary file 2 [file Image9.JPEG]

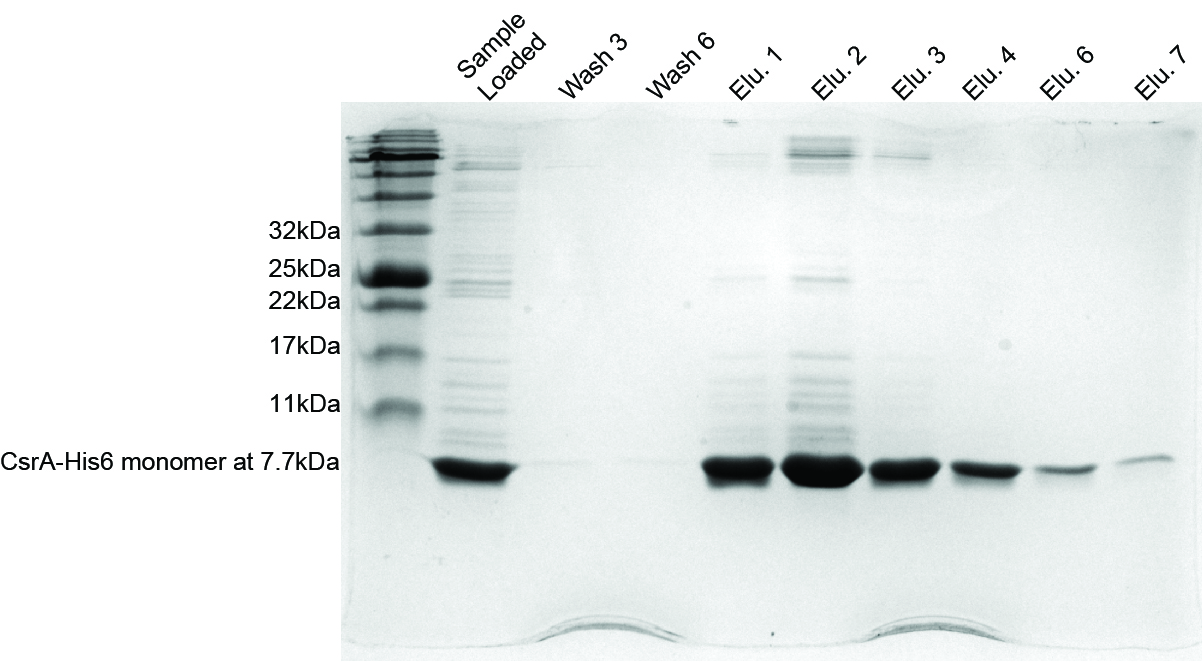

Supplement: Supplementary file 3 [file Image1.JPEG]

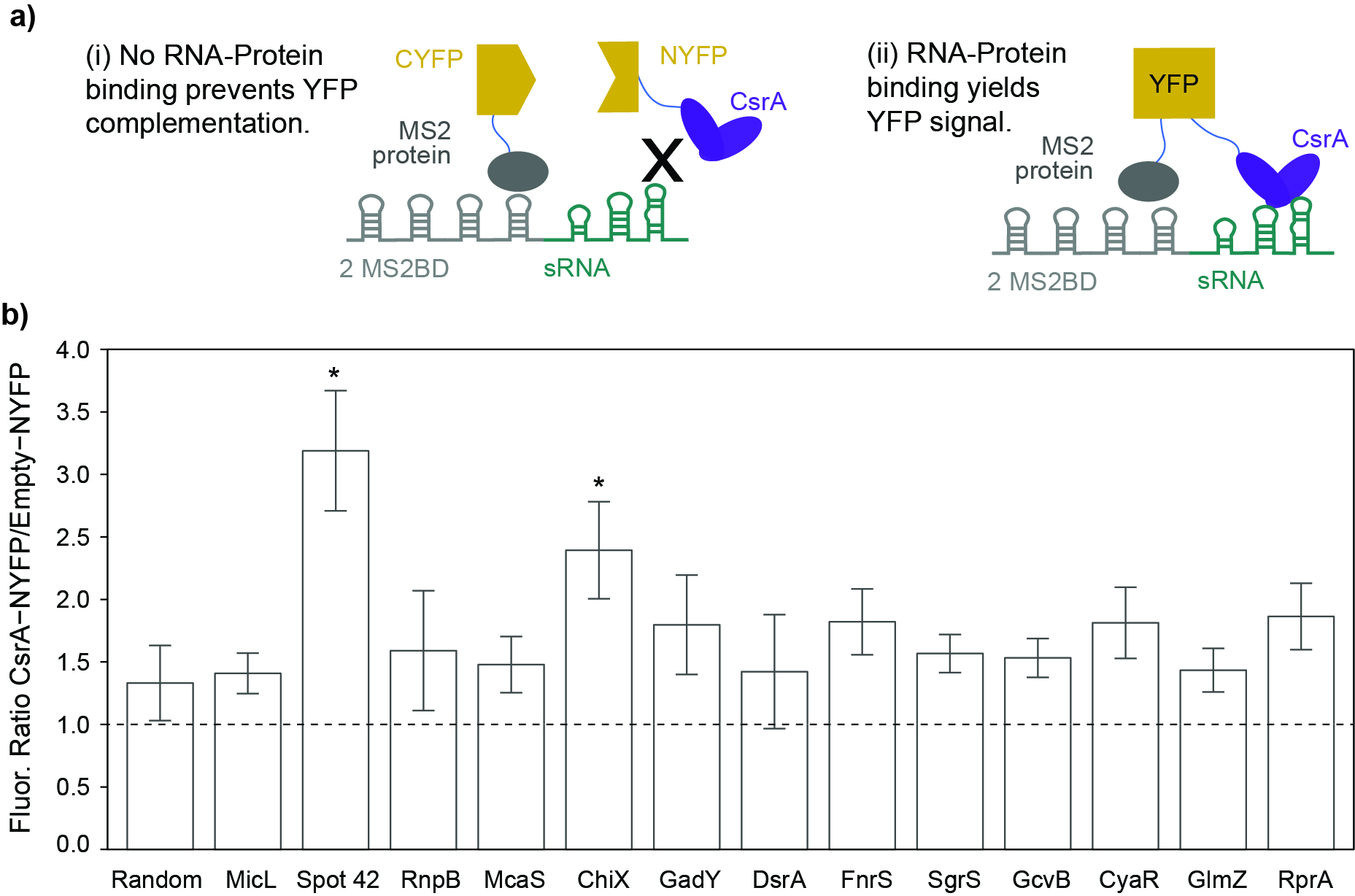

Supplement: Supplementary file 4 [file Image4.JPEG]

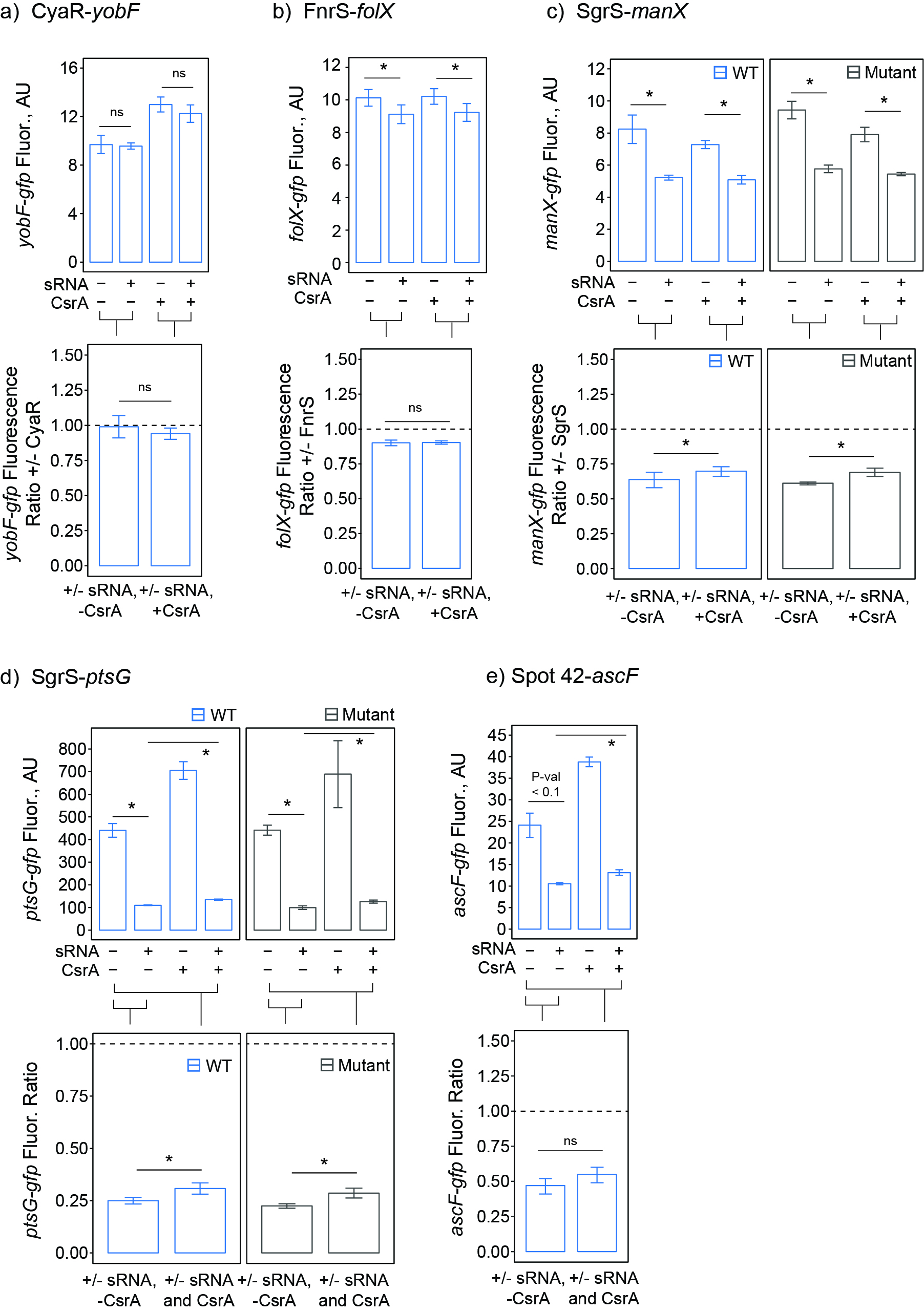

Supplement: Supplementary file 5 [file Image7.JPEG]

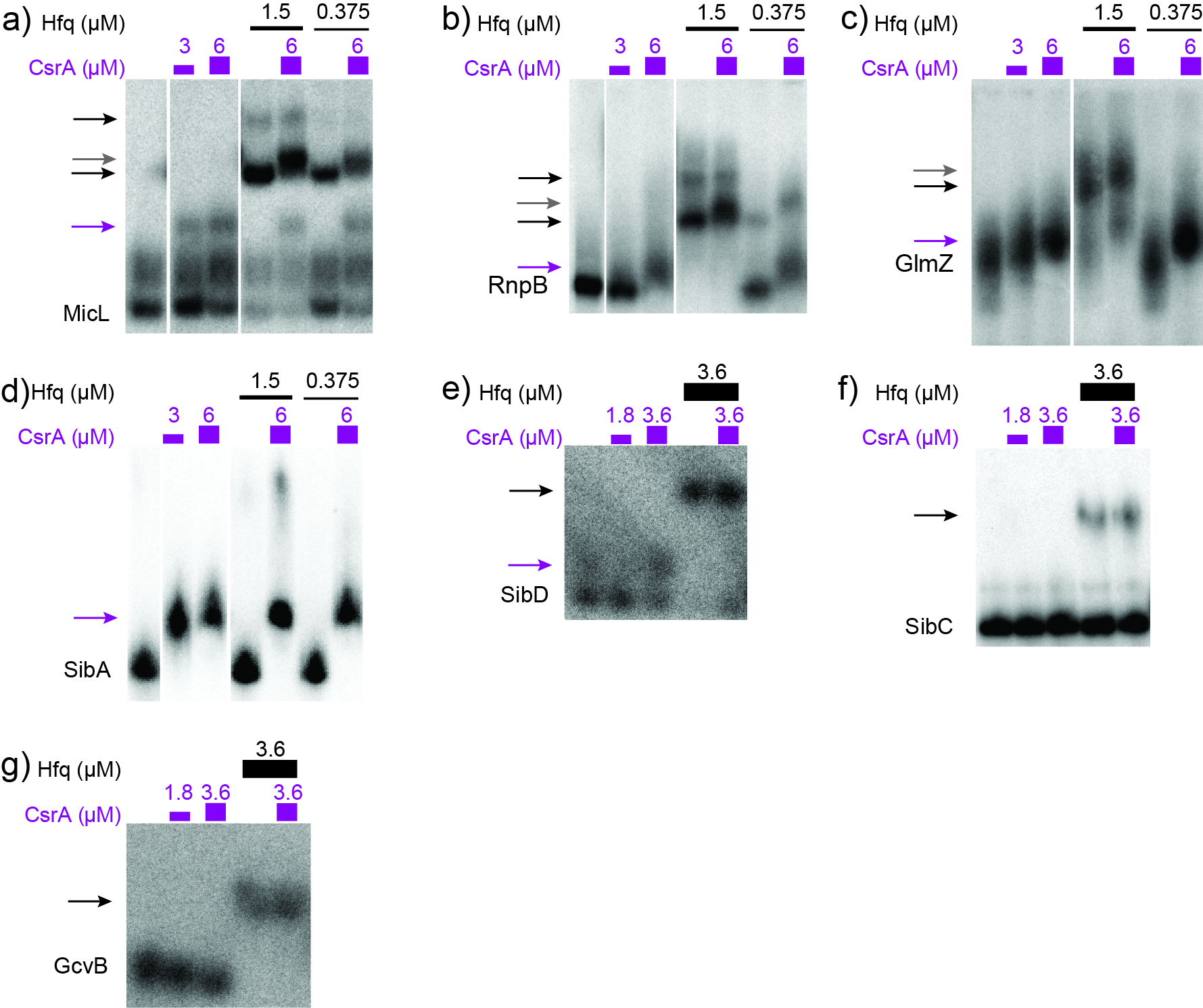

Supplement: Supplementary file 6 [file Image2.JPEG]

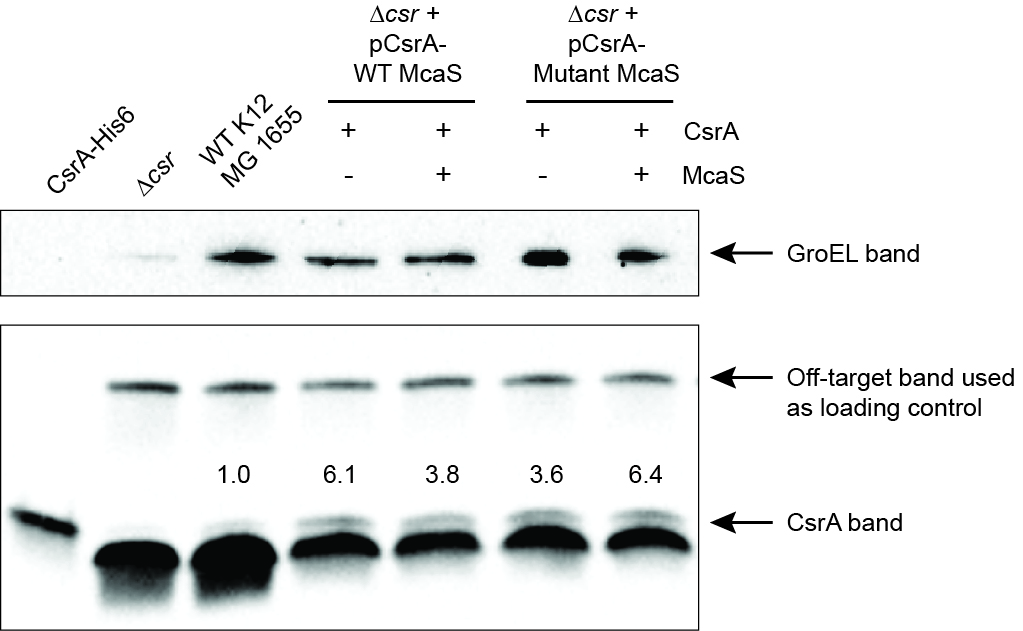

Supplement: Supplementary file 7 [file Image5.JPEG]

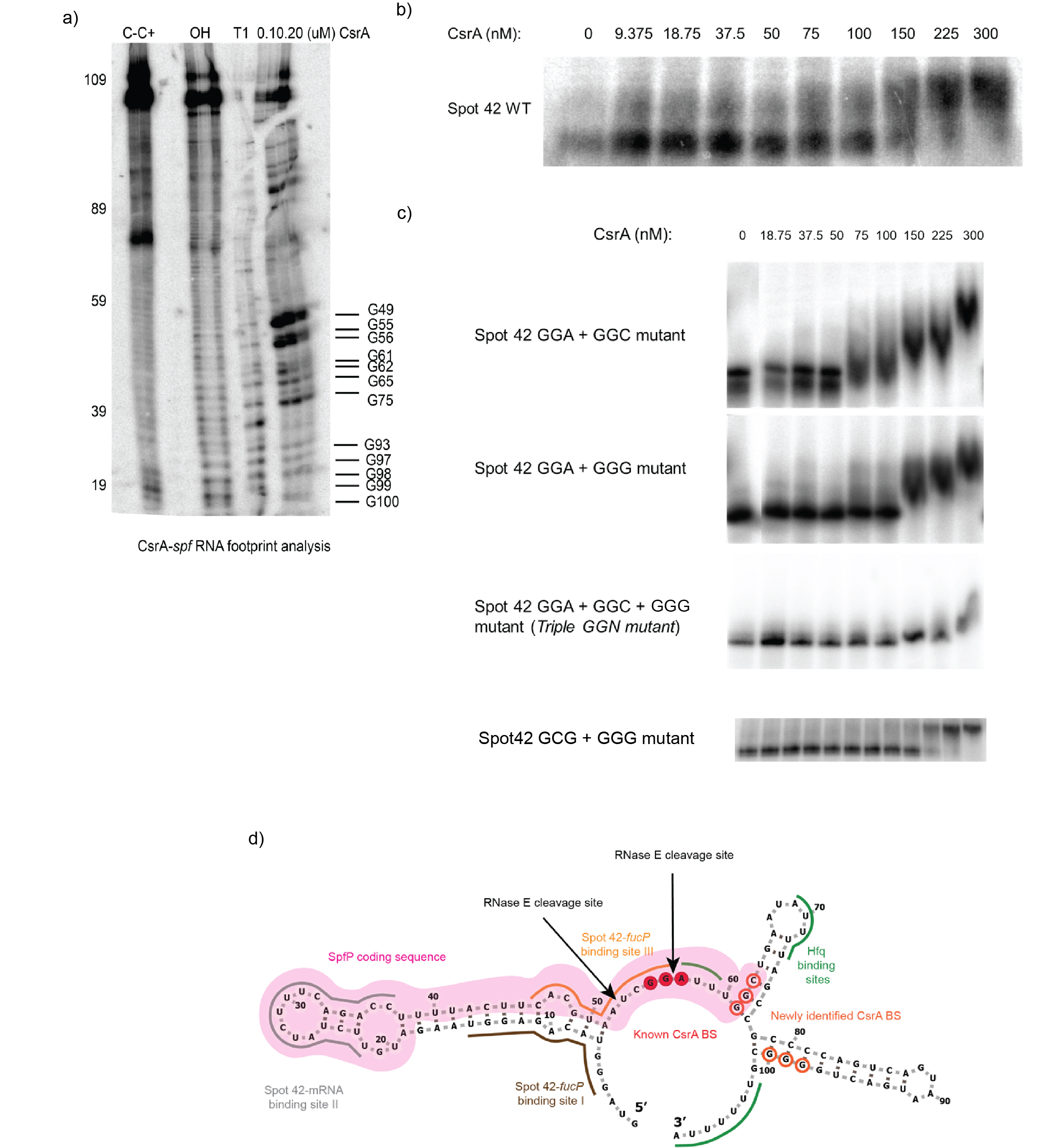

Supplement: Supplementary file 8 [file Image10.JPEG]

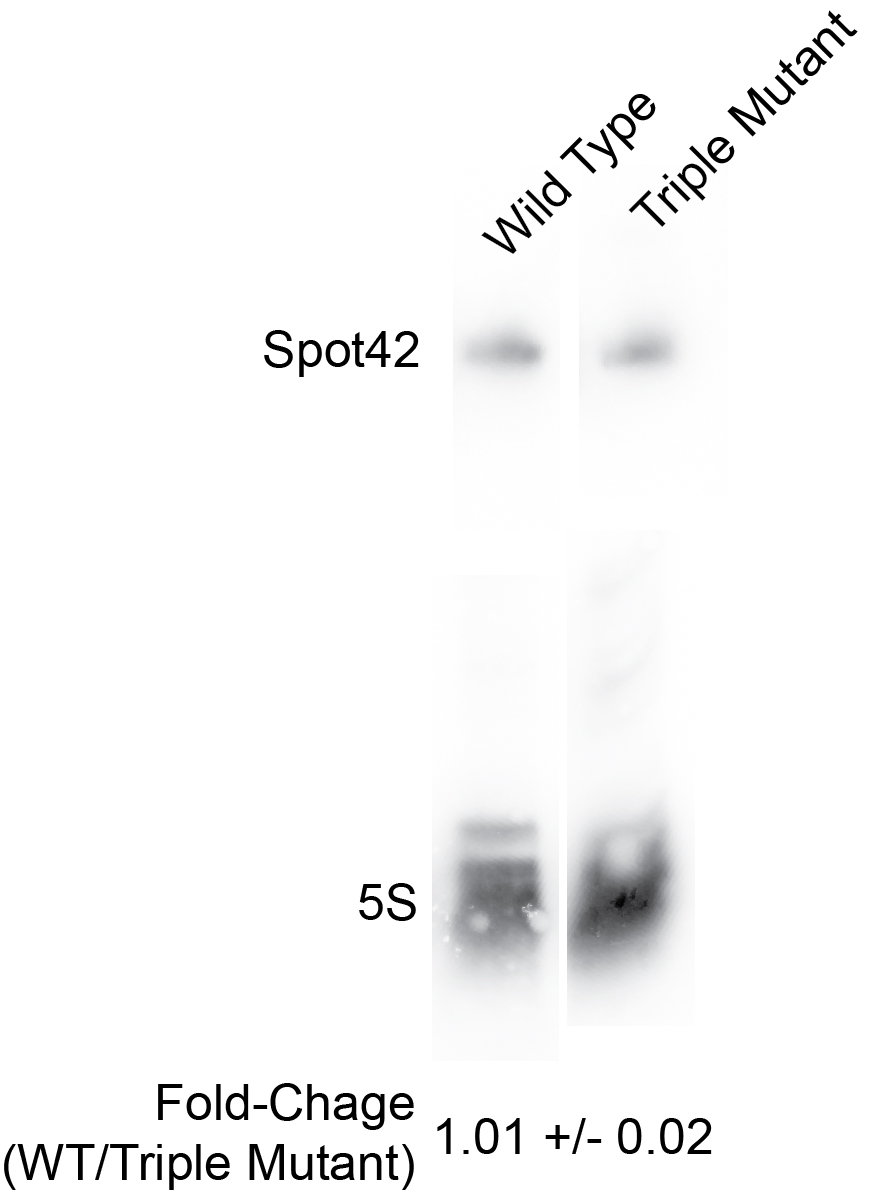

Supplement: Supplementary file 9 [file Image14.jpeg]

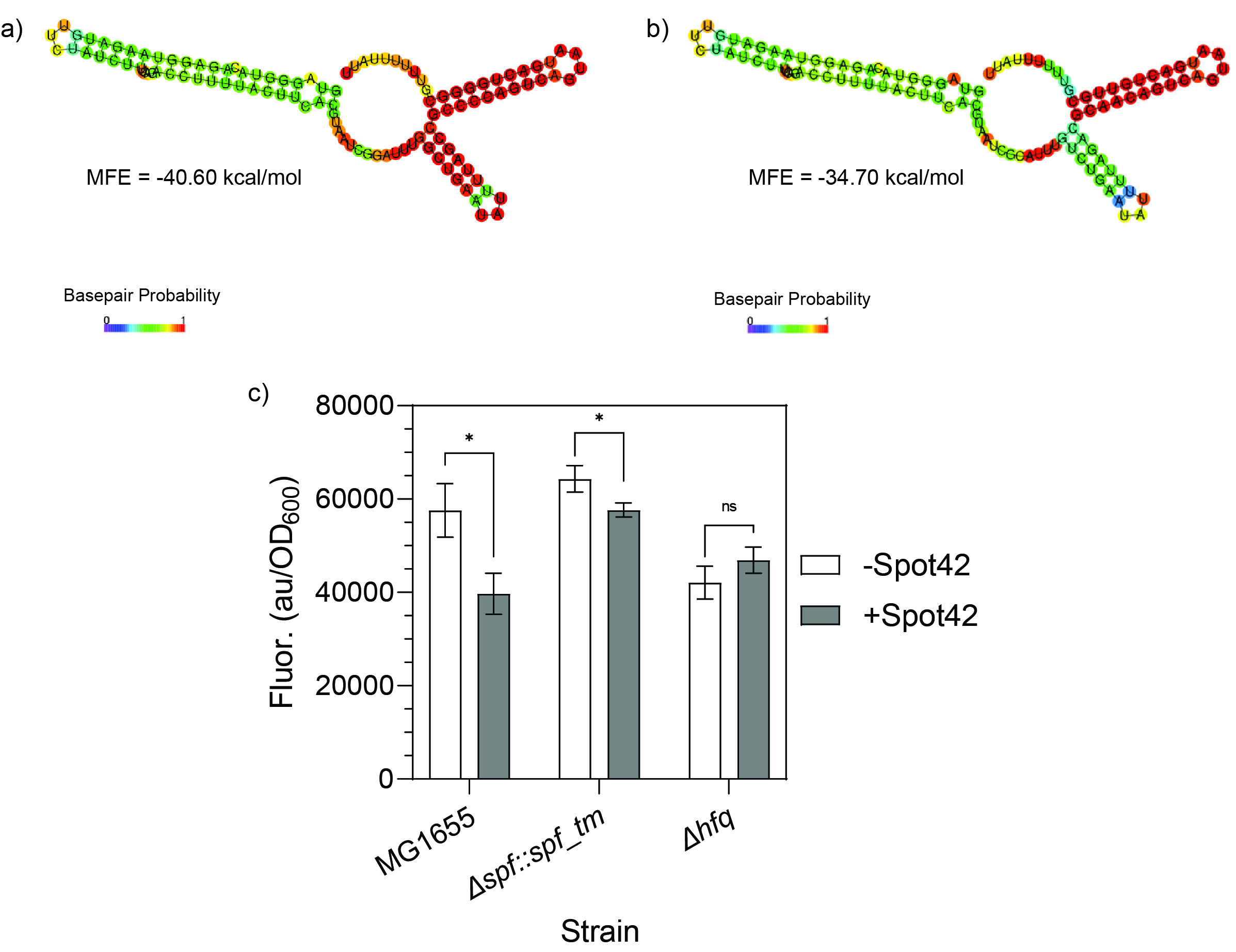

Supplement: Supplementary file 10 [file Image12.JPEG]

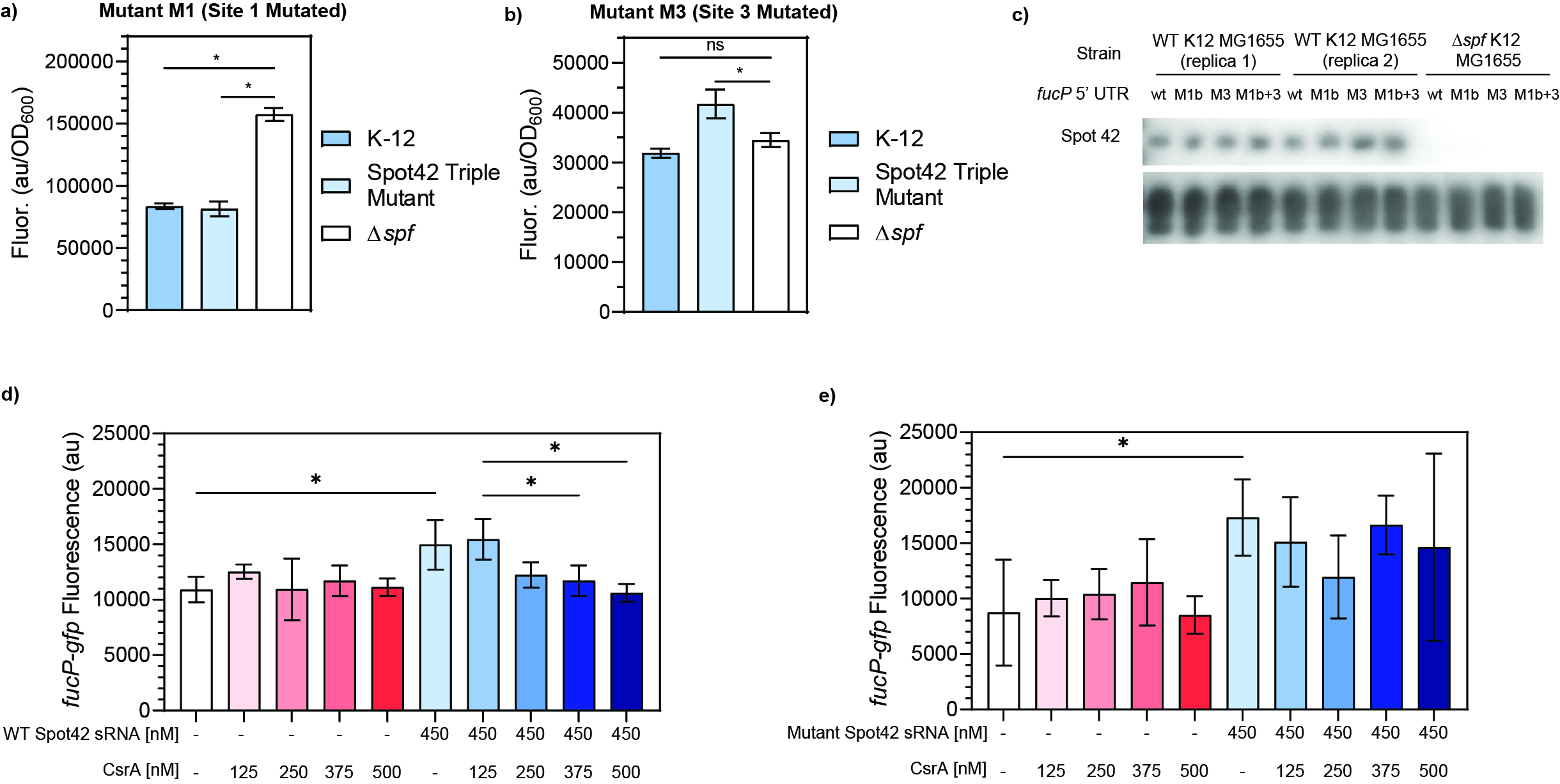

Supplement: Supplementary file 11 [file Image11.JPEG]

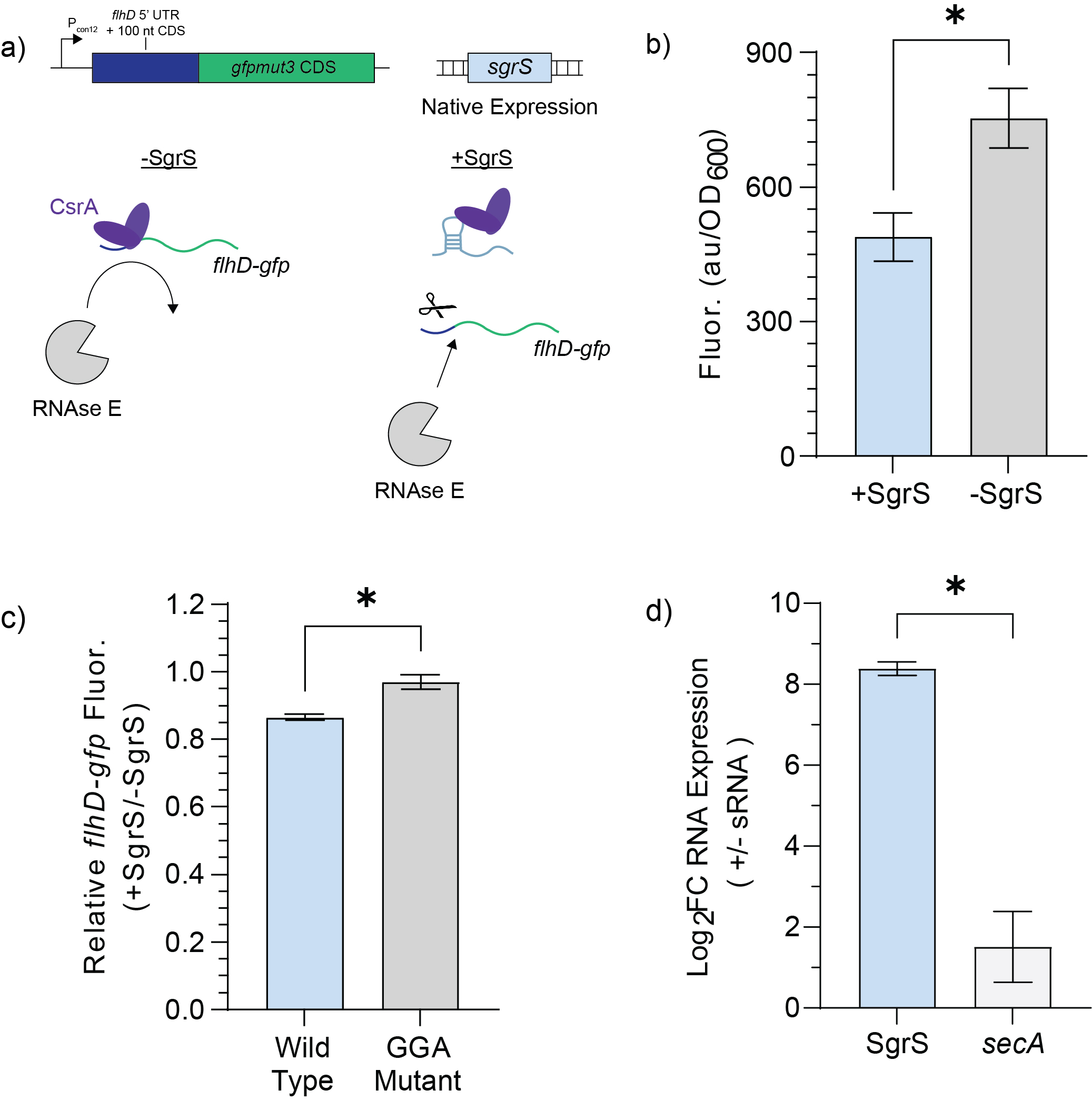

Supplement: Supplementary file 14 [file Image13.JPEG]

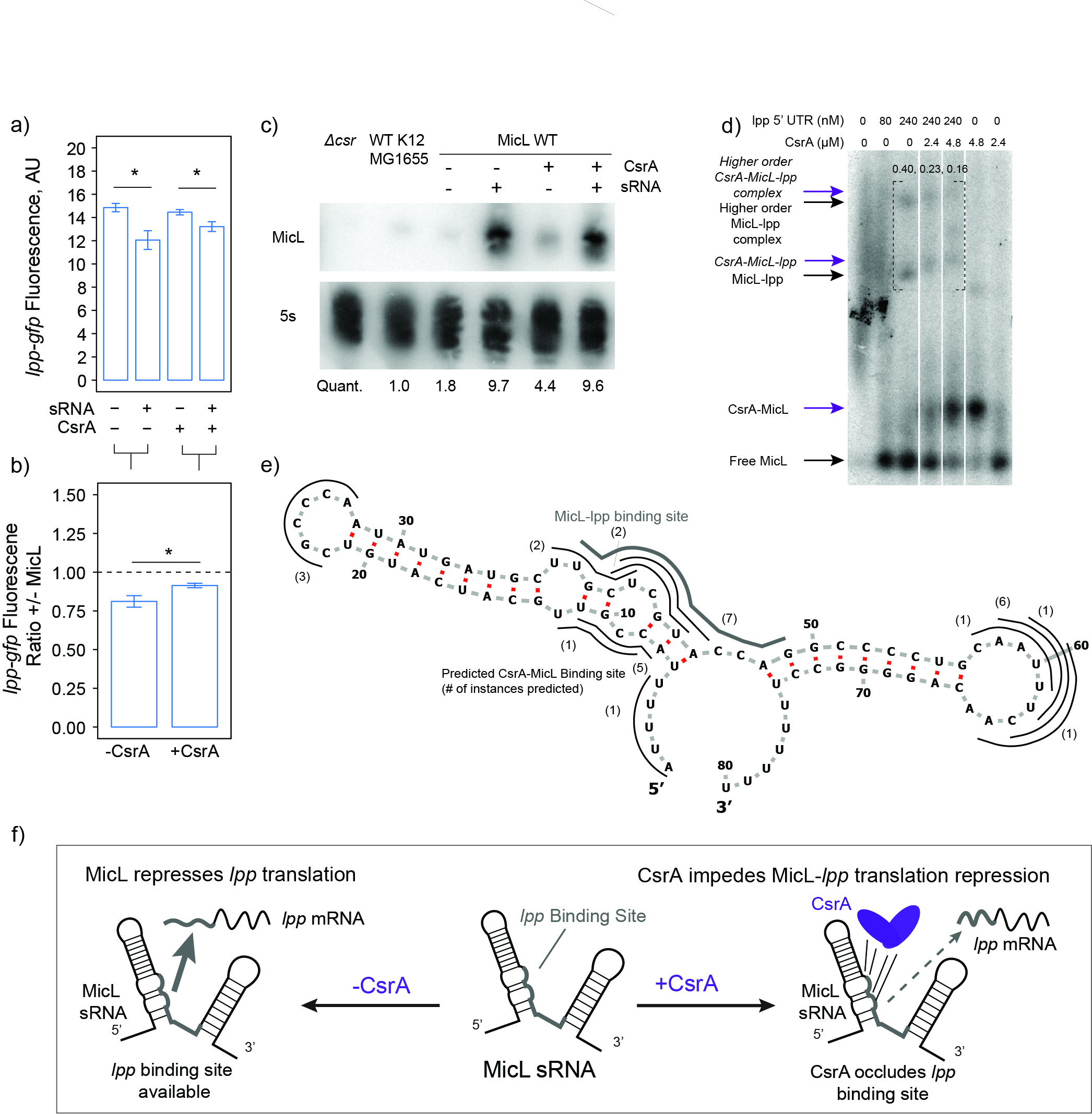

Supplement: Supplementary file 15 [file Image8.JPEG]

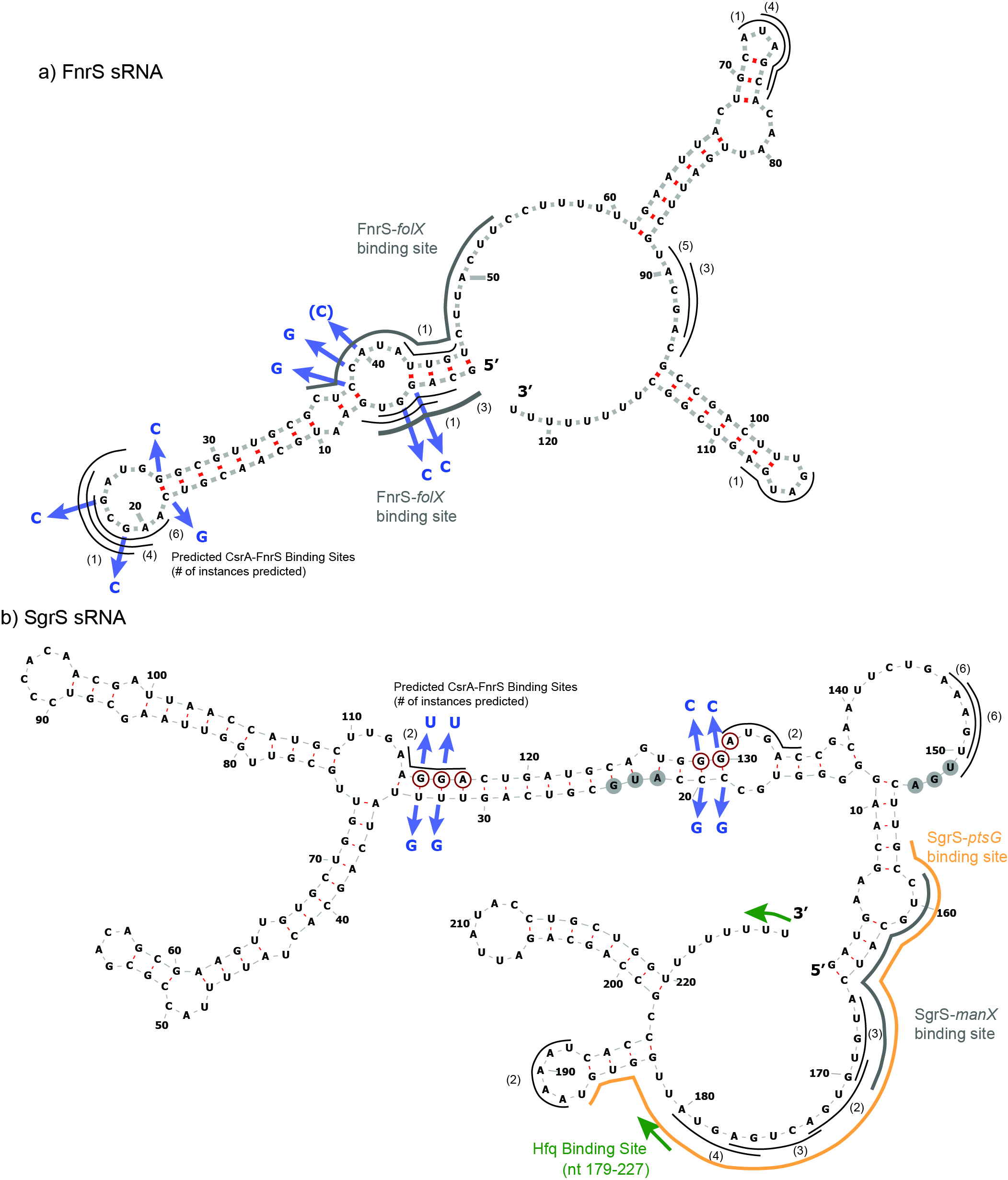

Supplement: Supplementary file 16 [file Image6.JPEG]
